# Prognostic factors for future mental, physical and urogenital health and work ability in women, 45–55 years: a six-year prospective longitudinal cohort study

**DOI:** 10.1186/s12905-020-01015-4

**Published:** 2020-08-12

**Authors:** Lena Rindner, Lena Nordeman, Gunilla Strömme, Irene Svenningsson, Åsa Premberg, Dominique Hange, Ronny Gunnarsson, Gun Rembeck

**Affiliations:** 1 Närhälsan, Södra Torget Health Care Center, Kvarngatan 4, SE-503 36 Borås, Sweden; 2 Region Västra Götaland, Research and Development Primary Health Care, Research and Development Center Södra Älvsborg, Borås, Sweden; 3 Primary Health Care, Public Health and Community Medicine, School of Public Health, Institute of Medicine, the Sahlgrenska Academy, University of Gothenburg, Gothenburg, Sweden; 4 Institute of Neuroscience and Physiology Department of Health and Rehabilitation, Unit of Physiotherapy, University of Gothenburg, Sahlgrenska Academy, Gothenburg, Sweden; 5 Region Västra Götaland, Research and Development Primary Health Care, Research and Development Center Fyrbodal, Gothenburg, Sweden; 6 Region Västra Götaland, Research and Development Primary Health Care, Research and Development Center Gothenburg, Gothenburg, Sweden; 7 Institute of Health and Care Sciences, University of Gothenburg, Sahlgrenska Academy, Gothenburg, Sweden; 8 Närhälsan, Svenljunga Health Care Center, Svenljunga, Sweden; 9 Närhälsan Borås Youth Centre, Region Västra Götaland, Borås, Sweden

**Keywords:** Menopause, women’s health, Workability, Sick leave, Social support, Primary health care, Mental health, Physical and urogential health

## Abstract

**Background:**

Impaired health due to stress is a common cause of long-term illness in women aged 45–55 years. It is a common cause for visits to primary health care (PHC) and may influence work-ability. The aim of this study was to investigate prognostic factors for future mental, physical and urogenital health as well as work-ability in a population of average women aged 45–55 years.

**Methods:**

This longitudinal cohort study initially assessed 142 women from PHC centers in southwestern Sweden. One houndred and ten accepted participation and were followed for 6 years. They were assessed using the self-reported questionnaires: the Menopause Rating Scale (MRS), the Montgomery-Asberg Depression Rating Scale (MADRS-S), the Short-Form Health Survey (SF-36). Descriptive data are presented of health, education, relationships and if they are working. Multicollinearity testing and logistic regression were used to test the explanatory variables.

**Result:**

Severity of symptoms in the MRS somatic and urogenital domains decreased while they increased in the psychological and depressive domains. Having tertiary education was associated with decreased overall mental health, vitality and social role functioning. Living with a partner was associated with increased physical role functioning, social role functioning and emotional role functioning.

**Conclusion:**

Quality of life seems to be enhanced by a good relationship with the partner, social support and work/life balance. Therefore, to improve women health women should early discuss ways in which these issues can be incorporated as they pursue their academic or career goals. Hence, we emphasize the importance of supporting women to gain increased awareness about a healthy life balance and to have realistic goals in work as well as in their social life.

## Background

Impaired health due to various forms of mental stress is a common cause of long-term illness in women in the age 45–55 years and a common cause of visits to primary health care (PHC) [1, 2]. Women suffer from long-term sickness and poor health to a greater degree than men [3]. Furthermore, women’s physical and mental health in Sweden shows a marked decrease around the ages 45–55 years [3]. During this phase in life, which often coincides with menopause, women undergo a hormonal conversion with reduced levels of oestrogen as well as bio-psyho-social changes [4–6].

The peri-menopausal period means the time around the menopause and also include the final menstrual period (FMP) [5]. The average age of FMP differs between women globally but women commonly reaches FMP in the ages 45–55 ([5, 6]. This period is often linked with symptoms from the vasomotor system, cardiovascular system, the skeleton, joints, muscles and urogenital tract [5–7]. Mental illness, particularly depressive symptoms, also show a marked increase during the ages 45–55 [8]. This phase in womens’ life has been labelled “the window of vulnerability” [9].

### Health

The World Health Organization (WHO) define health as “a state of complete physical, mental, and social well-being and not merely the absence of disease or infirmity”. Furthermore they define mental health as:” a state of well-being in which the individual realizes his or her own abilities, can cope with the normal stresses of life, can work productively and fruitfully, and is able to make a contribution to his or her community” [10].

### Women’s health: sick leave, prevalence and severity of symptoms

Adaptation disorders and stress reactions increase in Sweden as well as in other Organisation for Economic Co-operation and Development (OECD) countries and around one-third of working population suffers from poor mental health [1, 11]. The number of women on sick leave increased in Sweden from 58,000 to 99,000 between 2010 and 2015 which correspond to an increase of 71% psychiatric diagnoses increased the most and accounted for 59% of the increase in Sweden [1]. Stress, somatic symptoms, poor mental health and unhealthy relationships has become a very common cause for sick leave among women “in the prime of life” [3, 7, 12]. Good health and social support appear to be important prognostic factors for coping ability and having a future high quality of life [13, 14].

An important coping mechanism is that a women can identity their resources and use these to meet requirements and handle stress. It also include awareness of psychosocial resources in the woman’s surroundings. Differences in the ability to manage resources and social support may explain why some women exposed to stress don’t experience poor mental health [15].

The most prevalent and severe symptoms in women aged 45–55 years are muscle pain, sleep disorders, physical and mental fatigue, depression, sexual problems and characteristic hot flashes [7, 12]. Prognostic factors such as age, menopausal status, chronic diseases and sociodemographic characteristics, social support, income and educational level are associated with the frequency and severity of these symptoms [7, 12].

It should be noted that increased symptoms in this age group may not necessarily be correlated to a change in oestrogen levels [16]. Many of these issues involve the normal changes in this phase of life, but cause much concern and increased illness for some women [16]. Hence, mental health, social relations as well as income, working conditions and critical life events all seem to be related to each other [2, 10, 12, 14].

### Work ability

Participation in work are an important part of life and are essential for health and wellbeing. Physical, mental and urogenital symptoms in the age range 45–55 years are negatively associated with work ability [17]. Inability to work are more common (OR 8.4, 95% CI 4.1–17) in women suffering from mental, somatic and urogenital complaints compared to women not experiencing such discomfort [17].

High work-related stress combined with a large unpaid work load in the household increases the risk for both long term and short term sick leave [2, 18]. The association between partner relationships and sick leave have impact on the health and the ability to return to work [14]. A supportive partner relation may act as a buffer and counteract the effect of negative work-related stress [14]. Hence, important resources to increase return to work for women on long-term sick-leave can often be found in circumstances outside work, such as supportive relationships. Social support from co-workers, a healthy working relationships and good leadership styles of managers are of course also important facilitators for return to work [15].

### The remaining dilemma

The increasing number of long-term sick leave, ill health, increased risk for various diseases and increased number of visits to PHC among women in the age 45–55 years indicate the importance of specifically studying why the health of these women is deteriorating.

Conventional risk factors such as diabetes, hypertension, coronary artery disease or cardiac arrhythmia is, as for men and women in any age, also correlated with mortality in middle-aged women [19]. Serious physical and mental stress perceived to be related to work, family and homework indicated an increased risk of prolonged sick-leave in the working population [15, 20].

Risk factors for future poor health in women have in previous studies focused primarily on a wider age range than 45–55 years [15] and in both men and women combined [2]. Moreover, with a short follow up time [20], focused on a special work places [15], conducted in low-income countries [19] or did not focus on patients attending PHC [15, 19, 20]. Furthermore, most previous studies focused on women with various specific chronic diseases or with specific risk factors and have often included lifestyle interventions with or without drug administrations and they did not take place in PHC [19]. To our knowledge, no previous study focused on a long-term follow-up of a population of a population of average women 45–55 year.

This study aims to investigate prognostic factors for long-term future mental, physical and urogenital health as well as work ability in a population of average women aged 45–55 year.

## Methods

### Study design and selection of patients

This study was a 6-year longitudinal cohort study to evaluate prognostic factors for future work-ability and health of middle-aged women attending PHC. One hundred and forty-two patients were previously invited to a cross-sectional study [21] with a following randomized controlled clinical trial [22]. Six years later they were asked to participate in a second assessment.

The study was approved by the Regional Ethical Review Board in Gothenburg Sweden (registration number 041–09; T503–14). Written informed consent was obtained from all participants and confidentiality was ensured.

The women were recruited from March 2009 until December 2010. Women that, for any reason, visited the PHC centers in two municipalities in southwestern Sweden were consecutively asked to participate in the study. All participants were given a description of the study and informed about the right to decline participation or to withdraw from participation. All women accepting participation and meeting the inclusion criteria were invited to enroll in the study. The inclusion criteria were: female gender, 45 to 55 years of age and fluently understanding Swedish. The exclusions criteria were: unwillingness to continue participation in the study and new onset of severe mental illness.

To broaden the information of these women’s health situation, the present study added questionnaires at the six-year follow-up including more variables, such as the occurrence of the number of sick leave days in the last 90 days, work-ability, quality of life, current medication for high blood pressure and cardiovascular health. The questionnaires were mailed home with a pre-paid return envelope. A reminder envelope was sent if no questionnaires were returned within 3 weeks.

### Data collection

Demographic data including age, educational level, family situation, working status/capacity, menopause status, health status, current medication for high blood pressure and cardiovascular health was obtained. Health status included perceived mental, physical and urogenital health obtained from self-administrated questionnaires; The Menopause Rating Scale (MRS), The Montgomery-Asberg Depression Rating Scale (MADRS-S) and The Short-Form Health Survey (SF-36). The questionnaires MRS and MADRS-S were used in the first and second assessment while SF-36 was used only in the second assessment.

### Working status/capacity and sick leave

The work status asked for was currently working/studying, sick leave full-time, sick leave part-time, disability pension (full-time), disability pension (part-time), unemployed full-time or unemployed part-time. Sick leave was measured with self-assessed work ability and number of days on sick leave during the preceding 90 days.

### Menopausal status

Menopausal status was asked for and defined according to the criteria of the Stages of Reproductive Aging Workshop as: premenopausal (women having regular menses), perimenopausal (irregularities > 7 days from their normal cycle) and postmenopausal (no menses in the last 12 months) [7].

### Cardiovascular history

Presence of known high blood pressure was asked for by presenting the following alternatives: never had high blood pressure, or had high blood pressure only during previous pregnancy, or think they previously have had hypertension unrelated to pregnancy, or currently have high blood pressure but does not take any medication for this, or have high blood pressure and is currently taking medication for this. Any previous history of myocardial infarction or cerebrovascular illness was also asked for.

### Menopause rating scale (MRS)

For evaluation of the prevalence and severity of menopausal symptoms the MRS, as developed by Heinemann and validated in Sweden, was used [23]. The MRS is a self-administrated questionnaire consisting of 11 items, divided into three subscales reflecting; somatic symptoms - hot flushes, chest discomfort (such as irregular heart rhythm or feeling extra heart beats), sleeping problems and muscle and joint problems; mental symptoms **-** depressive mood, irritability, anxiety and physical and mental exhaustion; and urogenital symptoms - sexual problems, bladder problems and vaginal dryness. Each item ranged from 0 (not present) to 4 (1 = mild; 2 = moderate; 3 = severe; 4 = very severe). The MRS total score is the sum of the scores obtained for each subscale. Values equal or above 9 (somatic), 7 (mental), 4 (urogenital), and 17 (total) were used to define severe menopausal symptoms [23]. The MRS total score and somatic, urogenital and mental subscale score were calculated separately.

### Montgomery-Asberg depression rating scale (MADRS-S)

For the assessment of depression the MADRS-S was used [24]. It consists of nine questions, each scored from 0 to 6, where higher score indicates more severe symptoms; 1) Apparent Sadness 2) Inner Tension 3) Reduced Sleep 4) Reduced Appetite 5) Concentration Difficulties 6) Lassitude 7) Inability to Feel 8) Pessimistic Thoughts and 9) Suicidal Thoughts. The total MADRS-S score, calculated according to the manual [24], was interpreted as follows; 0–6 no depression, 7–19 mild depression, 20–34 moderate depression, > 34 severe depression [24].

### The short-form health survey (SF-36)

To examine the overall health, physical and mental, the short-form health survey (SF-36) was used [25]. The questionnaire consists of 36 items, divided into eight subscales: Physical Functioning (PF), Role-Physical (RF), Bodily Pain (BP), Mental Health (MH), Role-Emotional (RE), Vitality (VT), General Health (GH) and Social Functioning (SF). Scores on the subscales are between 0 and 100, higher value indicates better perceived health. Physical Component Summary (PCS) and Mental Component Summary (MCS) represents an overall health index of physical or mental health (range 0–100).

### Statistics

Descriptive data are presented by means and standard deviation (SD), median and percentiles, number and percentages at baseline and 6 years follow-up according to data level.

For all eight subscales, of SF-36 a cut-off was introduced at the mean value for Swedish women aged 46–54 years [25]. Being equal to or higher than the mean was coded as 1 and being worse off coded as 0. Several multivariable logistic regressions were made to identify potential prognostic factors, one for each of the following dependent variables estimated at the 6 year follow-up: workability, presence of hypertension and the dichotomization of all eight sub-scales in SF-36.

Multicollinearity testing was made before logistic regression by exploring the value of tolerance and variance inflator factor (VIF) between independent variables. Independent explanatory variables from the baseline measurement in the logistic regression were: age, working, living with a partner, having children living at home, have completed an exam at tertiary level (highest completed education), being in any kind of paid work, any depression measured with MADRS ≥7, MRS somatic symptoms ≥9, MRS urogenital symptoms ≥4, MRS mental symptoms ≥7 and MRS total score ≥ 17, received active intervention (to adjust for any intervention given in the previous RCT). The level of significance was set to *P* < 0.05. The IBM SPSS Windows version 22 was used for statistical analyses.

## Results

**Fig. 1:**
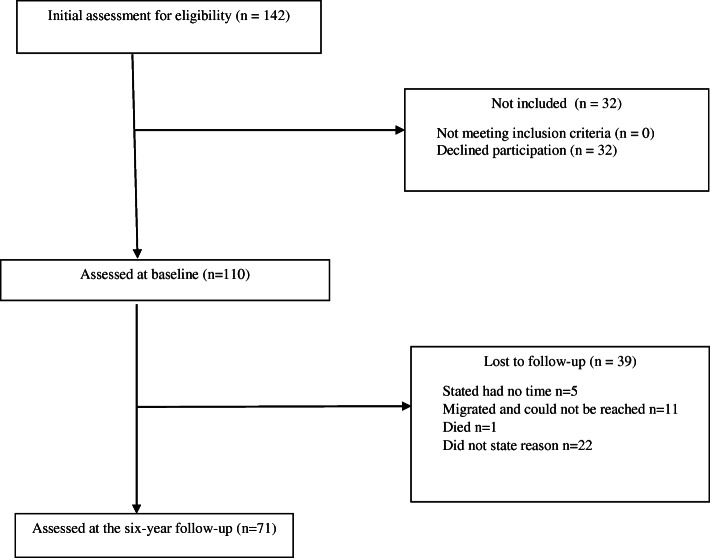
Participant flowchart

Sixty-five percent (*n* = 71/110) of the participants could be followed up after 6 years (Fig. 1). At baseline their average age was 50 years, most had an education of at least 10 years, were studying or working, living with a partner and 63% reported still menstruating (Table 1). Participants had moderate MRS mental and urogenital symptoms while the MRS somatic symptoms showed mild severity at baseline (Table 1). Information about menstruation was missing for 16 women due to 13 still using contraceptive treatments with hormones, one had a previous hysterectomy and two did not provide a clear statement on menstruation.

**Table 1:** Participant Characteristics at First Assessment (*n* = 71)

	Mean (SD)	Median (IR)	N (%)
Age (y)^a^	50 (3.1)		
Education (y)^b^			
Primary school (≤ 9)			15 (21)
Secondary school (10–12)			31 (44)
Tertiary school (> 12)			25 (35)
Work status/ Employment status^b^			
Currently working/studying^c^			61 (86)
Sick leave full-time			0 (0.0)
Sick leave part-time			0 (0.0)
Disability pension (full-time)			3 (4.0)
Disability pension (part-time)			2 (3.0)
Unemployed full-time			5 (7.0)
Unemployed part-time			0 (0.0)
Family status^b^			
Living with a partner			66 (93)
Children at home			17 (24)
Still menstruating^b,d^			34 (63)
Average MRS score^e^			
Somatic	4.3 (3.0)	4.0 (2–6)	
Urogenital	2.4 (2.5)	2.0 (0–4)	
Psychological	3.7 (3.1)	3.0 (1–6)	
Total MRS	10 (6.9)	9.0 (0–15)	
Average depression score^f,g^	7.3 (5.5)	6.0 (3–11)	

^a^First figure mean value (SD)

^b^n (%)

^c^Work more than 1 h a w = work more than one hour per week

^d^34/54 are still menstruating. Information is missing in 17 women

^e^Subscale and total Menopause Rating Scale (MRS) scoring. First figure mean (SD) second figure median (25th and 75th percentile). Degree of severity of the MRS and its domains indicated; Psychological domain; No, little (0–1), Mild (2–3), Moderate (4–6), Severe (7+), Somatic domain; No, little (0–2), Mild (3–4), Moderate (5–8), Severe (9+), Urogenital domain; No, little (0), Mild (1), Moderate (2–3), Severe (4+), Total score; No, little (0–4), Mild (5–8), Moderate (9–16), Severe (17+)

^f^Montgomery-Asberg Depression Rating Scale (MADRS) scoring. First figure mean (SD) second figure median (25th and 75th percentile). International standards; 0–6 p no depression, 7–19 p, mild depression, 20–34 p moderate depression, > 34 p severe depression

^g^Information is missing in 5 women (66/71)

### Changes from baseline to the 6-year follow-up

No women were on sick leave at baseline while four women stated they were on part time sick leave at the 6 year follow up. None of these stated the number of days on sick leave. A decrease in severity of total MRS score, somatic symptoms and urogenital symptoms was seen while mental and depressive symptoms increased (Table 2). As expected, the proportion of women having children living at home decreased by 59%.

**Table 2:** Changes from baseline to the 6-year follow-up (*n* = 71)

Family status			
Children at home	−59%		
Being in work^a^	−3%		
Menopausal Symptoms^b^			
Somatic	−0.23	(2.5)	
Urogenital	−0.52	(2.5)	
Psychological	+ 0.52	(3.0)	
Total MRS	−3.5	(5.6)	
Depressive Symptoms^c,d^	+ 0.38	(4.3)	

^a^Work more than 1 h a w = work more than one hour per week

^b^Menopause Rating Scale (MRS) subscale: Somatic symptoms - hot flushes, heart discomfort, sleeping problems and muscle and joint problems, Psychological symptoms **-** depressive mood, irritability, anxiety and physical and mental exhaustion, Urogenital symptoms - sexual problems, bladder problems and vaginal dryness, Total score **-** all subscales added. Higher score indicates more severe symptoms. Values are mean change (standard deviation)

^c^Montgomery-Asberg Depression Rating Scale (MADRS) scoring. Higher score indicates more severe symptoms

^d^Information is missing in 5 women (66/71)

### Cardiovascular symptoms at the 6-year follow-up

Current medication for high blood pressure was reported at the follow-up by 28/71 women. Another three women reported having high blood pressure but was not taking any medication. Four women described they have had high blood pressure in connection with pregnancy. Myocardial infarction or cerebral hemorrhage were reported by 5 women.

### Baseline prognostic factors s for good health and work-ability at the 6 year follow-up

The lowest tolerance and the highest VIF for any independent variable was 0.32 and 3.1 respectively. Hence, multicollinearity was not deemed to be a problem in any regression. Having tertiary education was associated with decreased overall mental health (MCS in SF 36) (Table 3), decreased vitality (VT) and social role functioning (SF) (Table 4). Living with a partner was associated with significantly increased physical role functioning (RF), social role functioning (SF) (Table 4) and emotional role functioning (RE) (Table 5).

**Table 3:** Predictors for good health, workability and presence of hypertension at 6 year follow-up

	Mental Health^a^		Physical Health^a^		Work ability		Hypertension^b^	
	(*n* = 69) MCS SF36 ≥ 50		(n = 69) PCS SF36 ≥ 50^b^		(n = 71)		(n = 71)	
Predictors	*p*-value	Effect size^c^	*p*-value	Effect size^c^	p-value	Effect size^c^	p-value	Effect size^c^
Age^d^	**0.0056**	1.5 (1.1–1.9)	0.67	0.96 (0.78–1.2)	0.46	1.1 (0.83–1.5)	0.13	1.2 (0.96–1.4)
Tertiary education	**0.019**	0.16 (0.034–0.74)	0.54	0.65 (0.17–2.6)	0.73	0.71 (0.10–4.8)	0.53	1.3 (0.40–4.4)
Work ability^e^	0.66	0.60 (0.061–5.8)	**0.017**	21 (1.7–250)	**0.0025**	51 (4.0–670)	**0.023**	0.12 (0.018–0.85)
Living with a partner	0.12	9.4 (0.56–160)	0.75	1.5 (0.13–17)	1.0	0.00 (0.00-.∞)	0.80	1.1 (0.10–13)
Children at home	0.61	1.6 (0.28–8.3)	0.41	2.0 (0.39–11)	0.47	2.5 (0.22–28)	0.26	0.51 (0.11–2.4)
Depression^f^	**0.027**	0.15 (0.027–0.81)	**0.012**	0.16 (0.037–0.67)	0.50	2.2(0.22–22)	0.88	1.1 (0.30–4.3)
MRS								
Somatic^g^	0.95	1.1 (0.090–13)	1.0	1.0 (0.12–8.4)	0.44	0.34 (0.22–5.3)	0.57	0.55 (0.071–4.2)
Psychologic^g^	**0.0069**	0.035 (0.0032–0.40)	0.93	1.1 (0.23–5.1)	0.065	0.11 (0.011–1.1)	0.27	2.0 (0.45–9.2)
Urogenital^g^	0.47	1.9 (0.34–10)	0.71	1.3 (0.32–5.3)	0.32	4.1 (0.26–12)	0.84	0.96 (0.25–3.7)
Active intervention^h^	0.66	0.74 (0.19–2.8)	0.99	1.0 (0.29–3.4)	0.54	1.8 (0.27–12)	0.26	2.0 (0.61–6.3)
Nagelkirke R square		0.53		0.36		0.49		0.26
Hosmer-Lemeshow test	0.88		0.35		0.29		0.49	
Area Under Curve^g^	< 0.001	0.87 (0.78–0.95)	< 0.001	0.81 (0.70–0.91)	< 0.001	0.87 (0.77–0.98)	< 0.001	0.76 (0.64–0.87)
Omnibus test of model	0.00006		0.019		0.0005		0.12	

^a^Cut of norm for Swedish women 45–54 years, SF36

^b^The part of the women stated having hypertension

^c^Effect size is Odds Ratio and (95% CI**)** for all predictors. First figure is p-value, second figure is predicted probability and CI, the odds ratio increase in score value

^d^Odds Ratio for an increase in age of 1 year between 45 and 55 years

^e^Working at least one hour/week

^f^Montgomery-Asberg Depression Rating Scale (MADRS) score ≥ 7 indicating at least mild depression. Information is missing in 5 women (66/71)

^g^Menopause Rating Scale and total Menopause Rating Scale (MRS) scoring. Somatic symptoms - hot flushes, heart discomfort, sleeping problems and muscle and joint problems; Psychological symptoms - depressive mood, irritability, anxiety and physical and mental exhaustion; Urogenital symptoms - sexual problems, bladder problems and vaginal dryness. Higher score indicates more severe symptoms

^h^The active group intervention is just as an adjustment and it is not the focus of this study

**Table 4:** Prognostic factors for good health estimated by the SF36 subscales Role Function, Physical Function, Vitality and Social Function at 6 year follow-up

	RF n = 71 ≥ 84^a^		PF ***n*** = 70 ≥ 86^a^		VT n = 70 ≥ 68^a^		SF n = 70 ≥ 88^a^	
Predictors	P-value	Effect size^b^	P-value	Effect size^b^	P-value	Effect size^b^	P-value	Effect size^b^
Age^c^	0.60	1.06 (0.86–1.3)	1.0	1.0 (0.83–1.2)	0.12	1.3 (0.94–1.7)	0.078	0.12 (0.98–1.5)
Tertiary education	0.12	0.34 (0.089–1.3)	0.78	0.83 (0.22–3.1)	**0.033**	0.18 (0.035–0.87)	**0.0051**	0.12 (0.28–0.50)
Work ability^d^	0.065	7.3 (0.89–59)	**0.028**	8.7 (1.3–61)	0.21	5.5 (0.39–80)	0.62	1.6 (0.24–11)
Living with a partner	**0.016**	32 (1.9–530)	0.87	1.2 (0.12–11)	0.60	0.47 (0.027–8.1)	**0.031**	21 (1.3–320)
Children at home	0.76	0.78 (0.16–3)	0.19	3.0 (0.59–15)	0.15	4.0 (0.61–26)	0.34	0.46 (0.91–2.3)
Depression^e^	0.077	0.28 (0.065–1.2)	**0.021**	0.20 (0.050–0.78)	**0.014**	0.092 (0.014–0.61)	0.11	0.31 (0.072–1.3)
MRS symptoms								
Somatic^f^	0.51	0.45 (0.043–4.7)	0.96	0.96 (0.15–6.2)	0.47	2.6 (0.19–36)	0.23	0.25 (0.025–2.4)
Psychologic^f^	0.45	0.56 (0.12–2).5)	0.88	1.1 (0.25–5.2)	1.0	0.00 (0.00-∞)	0.25	0.40 (0.085–1.9)
Urogenital^f^	0.65	1.4 (0.32–6.2)	0.47	1.7 (0.42–6.6	0.64	1.5 (0.26–8.7)	0.29	0.46 (0.11–2.0)
Active intervention^g^	0.059	3.0 (0.85–1.04)	0.14	2.6 (0.73–9.4)	0.10	3.4 (0.78–15)	0.065	0.31(0.87–1.1)
Nagelkirke R square		0.38		0.27		0.57		0.40
Hosmer-Lemeshow test	0.63		0.47		0.91		0.80	
Area Under Curve^f^	0.031	0.65 (0.52–0.78)	0.015	0.68 (0.54–0.81)	0.005	0.70 (0.58–0.83)	0.000	0.82 (0.72–0.91)
Omnibus test of model	0.009		0.12		< 0.001		0.006	

^a^Cut of for norm Swedish women age 46–54 years, SF36. SF36 subscales; Role function (RF), Physical function (PF), Vitality (VT) and Social function (SF). Higher score indicated better health

^b^Effect size is Odds Ratio and (95% CI**)** for all predictors. First figure is p-value, second figure is predicted probability and CI. The odds ratio increase in score value

^c^Odds Ratio for an increase in age of 1 year between 45 and 55 years

^d^Working more than one hour/week

^e^Montgomery-Asberg Depression Rating Scale (MADRS) score ≥ 7 indicating at least mild depression. Information is missing in 5 women (66/71)

^f^Menopause Rating Scale and total Menopause Rating Scale (MRS) scoring. Somatic symptoms - hot flushes, heart discomfort, sleeping problems and muscle and joint problems; Psychological symptoms - depressive mood, irritability, anxiety and physical and mental exhaustion; Urogenital symptoms - sexual problems, bladder problems and vaginal dryness. Higher score indicates more severe symptoms

^g^The active group intervention is just as an adjustment and it is not the focus of this study

**Table 5:** Prognostic factors for good health estimated by the SF36 subscales Mental health, General health, Role emotional and Bodily pain at 6 years follow-up

	MH n = 70 ≥ 80^a^		GH n = 70 ≥ 75^a^		RE n = 71 ≥ 87^a^		BP n = 69 ≥ 71^a^	
Predictors	p-value	Effect size^b^	p-value	Effect size^b^	p-value	Effect size^b^	p-value	Effect size^b^
Age^c^	0.11	1.4 (0.94–2.0)	0.071	1.2 (0.98–1.5)	0.062	1.2 (0.99–1.5)	0.93	0.99 (0.82–1.2)
Tertiary education	0.47	0.52 (0.092–3.0)	0.14	0.37 (0.10–1.4)	0.085	0.25 (0.050–1.2)	0.88	1.2 (0.33–3.6)
Work ability^d^	0.64	2.1 (0.093–48)	**0.025**	18 (1.4–220)	0.82	1.3 (0.16–10)	0.14	4.3 (0.62–29)
Living with a partner	0.74	1.9 (0.060–58)	0.12	8.3 (0.59–116)	**0.017**	29 (1.8–460)	0.43	0.37 (0.029–4.5)
Children at home	0.94	1.1 (0.094–13)	0.47	1.7 (0.39–7.6)	0.97	0.97 (0.18–5.3)	0.98	0.98 (0.22–4.4)
Depression^e^	0.16	0.18 (0.017–1.9)	0.13	0.33 (0.080–1.4)	0.24	0.38 (0.075–2.0)	0.062	0.30 (0.083–1.1)
MRS symptoms								
Somatic^f^	1.0	0.00 (0.00- ∞)	0.73	0.67 (0.070–6.5)	0.64	0.59 (0.061–1.9)	0.98	0.98 (0.14–7.1)
Psychologic^f^	1.0	0.00 (0.00-∞)	0.37	0.46 (0.084–2.5)	**0.011**	0.11 (0.020–0.61)	0.99	1.01 (0.22–4.6)
Urogenital^f^	0.44	2.1 (0.32–14)	0.52	0.63 (0.16–2.5)	0.74	1.3 (0.23–61)	0.75	1.2 (0.33–4.6)
Active intervention^g^	0.89	1.2 (0.24–5.2)	0.87	9.1 (0.34–3.6)	0.25	0.43 (0.10–1.8)	0.55	1.4 (0.46–4.3)
Nagelkirke R square		0.35		0.33		0.40		0.22
Hosmer & Lemeshow	0.94		0.33		0.47		0.98	
Area Under Curve^g^	0.001	0.84 (0.75–0.94)	< 0.001	0.79 (0.68–0.90)	< 0.001	0.85 (0.75–0.95)	0.001	0.72 (0.60–0.85)
Omnibus test of model	0.13		0.029		0.010		0.28	

^a^Cut of norm for Swedish women 45–54 years, SF36. SF36 Subscales; Mental health (MH), General health (GH), Role emotional (RE) and Bodily pain (BP). Higher score indicated better health

^b^Effect size is Odds Ratio and (95% CI**)** for all predictors. First figure is p-value, second figure is predicted probability and CI. The odds ratio increase in score value

^c^Odds Ratio for an increase in age of 1 year between 45 and 55 years

^d^Working more than one hour/week

^e^Montgomery-Asberg Depression Rating Scale (MADRS) score ≥ 7 indicating at least mild depression. Information is missing in 5 women (66/71)

^f^Menopause Rating Scale and total Menopause Rating Scale (MRS) scoring. Somatic symptoms - hot flushes, heart discomfort, sleeping problems and muscle and joint problems; Psychological symptoms - depressive mood, irritability, anxiety and physical and mental exhaustion; Urogenital symptoms - sexual problems, bladder problems and vaginal dryness. Higher score indicates more severe symptoms

^g^The active group intervention is just as an adjustment and it is not the focus of this study

## Discussion

This study showed that women in ages 45–55 years living with a partner appear to have a better chance for having good health 6 years later than those living as singles in physical role functioning (RF), social role functioning (SF) and emotional role functioning (RE) (Table 3, 4). Having a tertiary education was associated with a higher risk for decreased mental health (MCS in SF 36), vitality (VF) and social role functioning (SF) (Table 4). It appears that level of education might be an important aspect to take into consideration in women with poor mental health.

### Health and role-functioning

The role-functioning in SF-36 suggests that important factors to mental health is the ability to participate in social interaction in and outside the home as well as the ability to participate in work or other regular activities without being hampered by emotional problems [26]. In addition to role functioning a Swedish study showed that women in Sweden have higher level of education then men but the average income was lower. Women also experienced more anxiety and experienced their health as worse compared with men [2].

Social determinants of health are related to the extent the woman lives in an equally, friendly, harmless and safe environment with their partner. Other factors related to women’s health are the ability to cope and solve problems in life, if there is a balance in life between work and leisure and if there is time for recovery.

### Tertiary education

Historically we know people with shorter education more often suffer from mental health problems and experience their health as poor compared to those who have a longer education [18]. Hence, women with higher education have previously been noted to have lower sick leave than women with shorter education [18]. However, the last 5 to 10 years has seen a dramatic change where the largest increase in sick leave has been in the group with longer education [27]. High demands at work and home as well as psychosocial factors seems involved resulting in stress related diagnoses increasing more for academics than for women with shorter education [18].

Higher education for women will have benefits but may also be linked to higher exposure to certain risks, for example including patriarchal systems that hinder women’s progress in business and academia, excessive burdens from taking care of others, the tension between traditional administrations and realities in life and violence and sexual harassment in the workplace. Hence, higher education may be linked to a higher exposure to some risks, most of which may be improved through action from the state [2, 10]. A previous Swedish report showed a strong increase in long-term sick leave, mainly among female academics, where the most common problems were caused by stress-related illness and depression [27]. Our findings seem to support this showing that higher education was associated with future lower mental health, lower vitality and lower social role function. It is important for women to maintain awareness about a healthy balance in life and to have realistic goals in work as well as in their social life.

### Living with a partner

Pervious research showed that social support and to be happily married were important factors for good mental health with an association between marital status and good health [28]. Midlife happily married women reported better mental health and life satisfaction compared with women unhappy with their marriages and single women [28, 29]. This was further confirmed in a meta-analysis describing associations between marital status and physical health showing that a higher marital quality was associated with a better health and lower risk of mortality [28]. This study confirms these previous findings showing that living with a partner indicated a better chance for having a good health 6 years later compared to those living as singles in respect of physical role functioning (RF), social role functioning (SF) and emotional role functioning (RE). A key to good health may be effective communication in the partner relationship [29]. Hence, it is important to be aware of and consider asking about marital status and quality of the partner relation when discussing health issues with women in age 45–55 years.

### Strengths and limitations

The use of validated questionnaires such as MRS and SF-36 is a strength and allow comparisons with other studies. A potential limitation is that we did not include information about being pre and post-menopausal at baseline as a prognostic factor because we lacked information about this variable in 17 women (Table 1). Some women didn’t know or could not state if they were postmenopausal due to the use of contraceptives [30]. Another limitation is that we didn’t used the questionnaire SF-36 at the first assesmet.

## Conclusion

This six-year long cohort-study of women’s health in the age 45–55 years shows that living in a good relation with a partner seems to be a strong factor for good perceived health 6 years later while higher education seem be a risk factor for poorer mental health 6 years later. Hence, awareness needs to be raised that higher education, while being beneficial in some aspects, might also be linked to higher exposure to certain risks.

The preventive focus should be on social determinants and striving to live life as best as possible, ensure maintaining a social network, invest in having a good relationship with partners and continue to learning.

Hence, it is important to early discuss with women if the life they live are creating a lower quality of life for them. If so, an important subsequent discussion should be held about reevaluating the way they live and the need for change. This includes the partner relationship and social support structures. A reasonable conclusion would be that women in the age 45–55 years attending PHC should be supported to gain increased awareness about a healthy balance in life and to have realistic goals in work as well as in their social life.

## Data Availability

The authors make available the data set used for the study.

## Abbreviations

PHC: Primary health care
MRS: The menopause rating scale
MADRS-S: The montgomery-asberg depression rating scale
SF-36: The short-form health survey
PF: Physical functioning
RF: Role-physical
BP: Bodily pain
MH: Mental health
RE: Role-emotional
VT: Vitality
GH: General health
SF: Social functioning
PCS: Physical component summary
MCS: Mental component summary
SD: Standard deviation
VIF: Variance inflator factor
